# Supplementary material for: Novel Genotype of HA Clade 2.3.4.4b H5N8 Subtype High Pathogenicity Avian Influenza Virus Emerged at a Wintering Site of Migratory Birds in Japan, 2021/22 Winter
Source: Pathogens. 2024 May 2;13(5):380. doi: 10.3390/pathogens13050380 (PMC11124138; doi:10.3390/pathogens13050380)
Supplement: Supplementary file 1 [file pathogens-13-00380-s001.zip › pathogens-2975142-supplementary.pdf]

## Supplementary Files

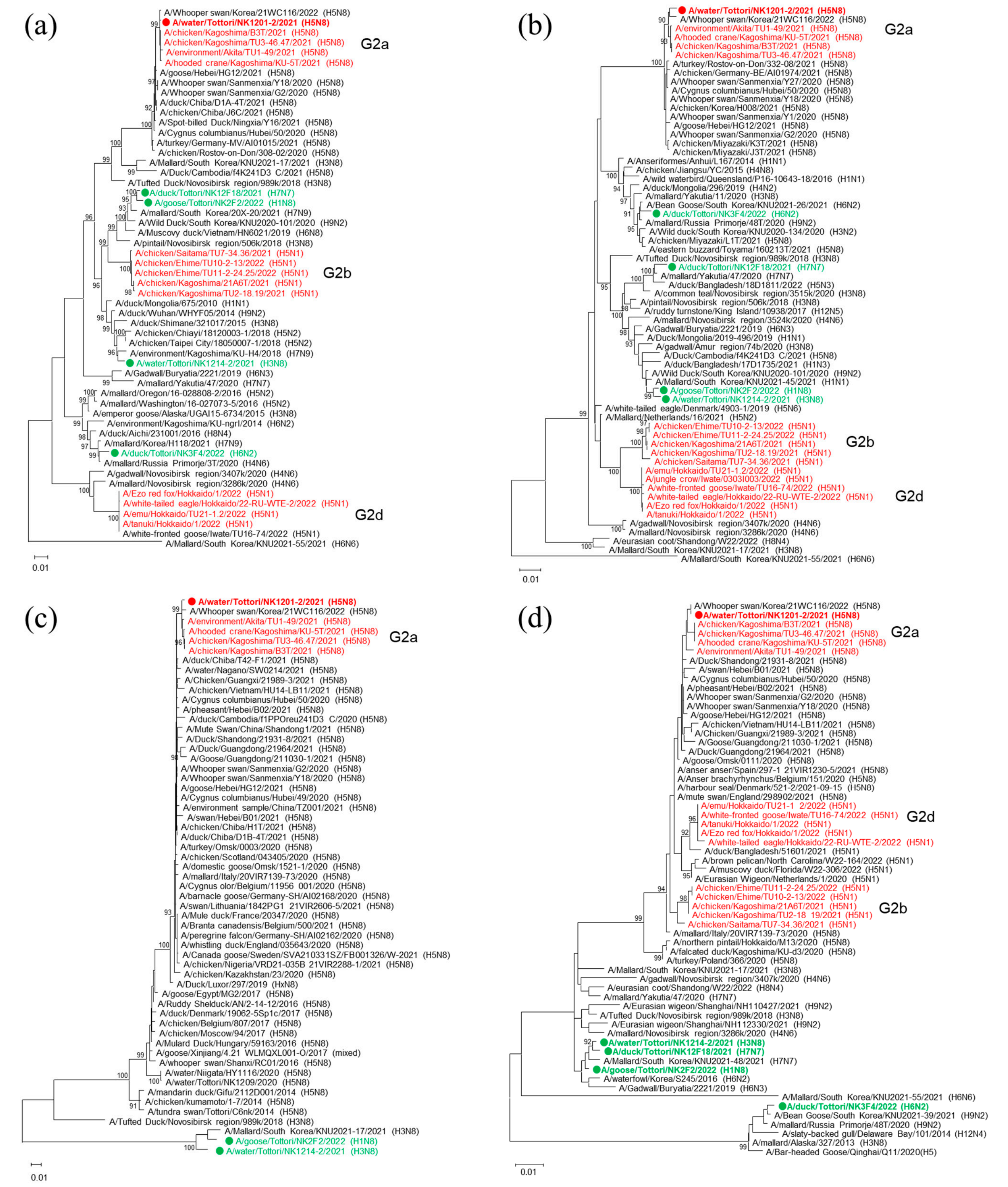

(e)

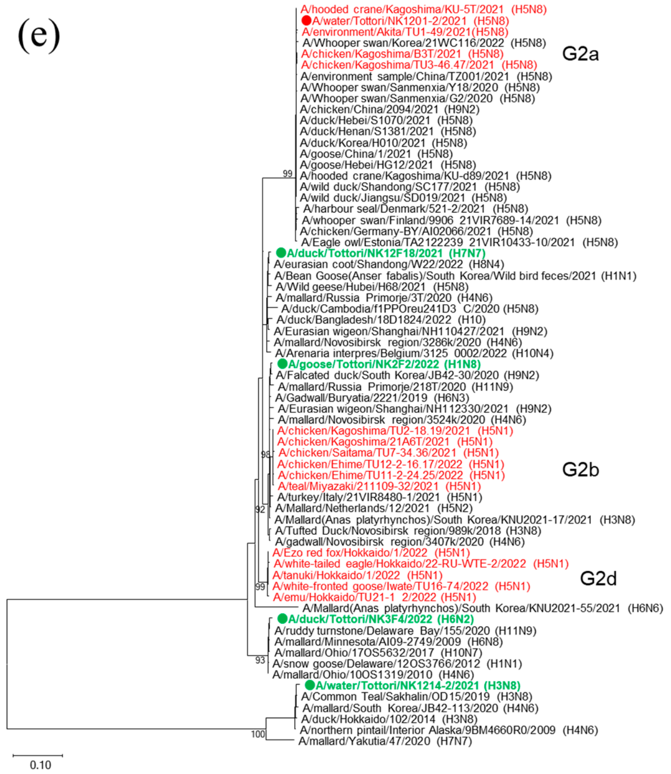


**Figure S1**. Phylogenetic trees based on coding region of five gene segments of NK1201 HPAIV and LPAIVs isolated at Nikko wintering site. PB2, 1-2280 bp (a); PA, 1-2151 bp (b); N8 NA, 1-1413 bp (c); M, 1-982 bp (d) and NS, 1-838 bp (e) trees were constructed using the maximum likelihood method with the general time-reversible model and 1,000 bootstrap replicates. G2a, G2b, and G2d indicate the classification of H5N8 and H5N1 HPAIVs isolated in the 2021-2022 winter season in Japan based on their HA gene sequences [19]. Bootstrap values >90% are shown at the nodes. Red: H5N8 HPAIVs isolated during 2021-2022 in Japan; Green: LPAIV isolates from wintering site for migratory birds in Tottori Prefecture. Filled circle indicate isolates in this study.

**Table S1**: Nucleotide and amino acid similarities of NK1201 genes to most closely related influenza viruses

| Genes | Closely related viruses | Collection date | Nucleotide identity (%) | Amino acid identity (%) |
|---|---|---|---|---|
| Polymerase basic 2 (PB2) | A/chicken/Kagoshima/TU3-46.47/2021 (H5N8) | 2021-11-15 | 2277/2280 (99.87) | 99.60 |
| | A/chicken/Kagoshima/B3T/2021 (H5N8) | 2021-11-14 | 2277/2280 (99.87) | 99.60 |
| | A/Whooper swan/Korea/21WC116/2022 (H5N8) | 2022-01-23 | 2276/2280 (99.82) | 99.60 |
| | A/hooded crane/Kagoshima/KU-5T/2021 (H5N8) | 2021-11-19 | 2275/2280 (99.78) | 99.60 |
| | A/environment/Akita/TU1-49/2021 (H5N8) | 2021-11-10 | 2273/2280 (99.69) | 99.60 |
| | A/mallard/Miyazaki/4501C605/2021 (H5N8) | 2021-01-24 | 2271/2280 (99.69) | 99.60 |
| Polymerase Acidic (PA) | A/hooded crane/Kagoshima/KU-5T/2021 (H5N8) | 2021-11-19 | 2139/2151 (99.44) | 99.86 |
| | A/chicken/Kagoshima/TU3-46.47/2021 (H5N8) | 2021-11-15 | 2139/2151 (99.44) | 99.86 |
| | A/environment/Akita/TU1-49/2021 (H5N8) | 2021-11-10 | 2139/2151 (99.44) | 99.86 |
| | A/chicken/Kagoshima/B3T/2021 (H5N8) | 2021-11-14 | 2139/2151 (99.44) | 99.86 |
| | A/Whooper swan/Sanmenxia/Y18/2020 (H5N8) | 2020-11-13 | 2138/2151 (99.40) | 99.86 |
| | A/Whooper swan/Sanmenxia/G2/2020 (H5N8) | 2020-11-04 | 2138/2151 (99.40) | 99.86 |
| | A/Whooper swan/Sanmenxia/Y1/2020 (H5N8) | 2020-10-30 | 2138/2151 (99.40) | 99.86 |
| | A/chicken/Korea/H008/2021 (H5N8) | 2021-01-04 | 2138/2151 (99.40) | 99.86 |
| | A/Whooper swan/Sanmenxia/Y27/2020 (H5N8) | 2020-11-16 | 2137/2151 (99.35) | 99.86 |
| | A/Whooper swan/Korea/21WC116/2022 (H5N8) | 2022-01-23 | 2136/2151 (99.30) | 99.86 |
| Hemagglutinin (HA) | A/Whooper swan/Korea/21WC116/2022 (H5N8) | 2022-01-23 | 1694/1704 (99.41) | 99.29 |
| | A/environment/Akita/TU1-49/2021 (H5N8) | 2021-11-10 | 1691/1704 (99.24) | 98.94 |
| | A/Whooper swan/Sanmenxia/H735/2020 (H5N8) | 2020-11-11 | 1691/1704 (99.24) | 98.94 |
| | A/goose/Hebei/HG12/2021 (H5N8) | 2021-01 (Day unknown) | 1691/1704 (99.24) | 98.94 |
| | A/Duck/Shandong/21232-5/2021 (H5N8) | 2021-03 (Day unknown) | 1691/1704 (99.24) | 98.94 |
| | A/Goose/Shandong/21153-3/2021 (H5N8) | 2021-02-01 | 1691/1704 (99.24) | 98.94 |
| | A/chicken/Akita/7C/2021 (H5N8) | 2021-11-09 | 1691/1704 (99.24) | 98.94 |
| | A/chicken/Korea/H544/2020 (H5N8) | 2020-12-29 | 1691/1704 (99.24) | 98.94 |
| | A/chicken/Kagoshima/TU3-46.47/2021 (H5N8) | 2021-11-15 | 1690/1704 (99.18) | 99.12 |
| | A/chicken/Kagoshima/B3T/2021 (H5N8) | 2021-11-14 | 1690/1704 (99.18) | 99.12 |
| | A/hooded crane/Kagoshima/KU-5T/2021 (H5N8) | 2021-11-19 | 1689/1704 (99.12) | 99.12 |
| Neuraminidase (NA) | A/environment/Akita/TU1-49/2021(H5N8) | 2021-11-10 | 1406/1413 (99.50) | 99.15 |
| | A/chicken/Kagoshima/TU3-46.47/2021 (H5N8) | 2021-11-15 | 1404/1413 (99.36) | 99.36 |
| | A/chicken/Kagoshima/B3T/2021 (H5N8) | 2021-11-14 | 1404/1413 (99.36) | 99.36 |
| | A/hooded crane/Kagoshima/KU-5T/2021 (H5N8) | 2021-11-19 | 1404/1413 (99.36) | 99.36 |
| | A/Whooper swan/Korea/21WC116/2022 (H5N8) | 2022-01-23 | 1403/1413 (99.29) | 98.94 |
| | A/Whooper swan/Sanmenxia/Y21/2020 (H5N8) | 2020-11-14 | 1401/1413 (99.15) | 98.94 |

**Table S1**: Nucleotide and amino acid similarities of NK1201 genes to most closely related influenza viruses (continued)

| | | | | |
|---|---|---|---|---|
| Matrix protein (M) | A/Whooper swan/Korea/21WC116/2022 (H5N8) | 2022-01-23 | 981/982 (99.90) | M1: 100<br>M2: 100 |
| | A/environment/Tokushima/T47-36/2021 (H5N8) | 2021-02-09 | 980/982 (99.80) | M1: 100<br>M2: 100 |
| | A/hooded crane/Kagoshima/KU-d89/2021 (H5N8) | 2021-01-16 | 980/982 (99.80) | M1: 100<br>M2: 100 |
| | A/chicken/China/JM01/2020 (H5N8) | 2020-12<br>(Day unknown) | 980/982 (99.80) | M1: 100<br>M2: 100 |
| | A/tundra swan/Shanghai/CM20111601/2020 (H5N8) | 2020-11-16 | 980/982 (99.80) | M1: 100<br>M2: 100 |
| | A/Whooper swan/Sanmenxia/Y25/2020 (H5N8) | 2020-11-18 | 980/982 (99.80) | M1: 100<br>M2: 100 |
| | A/environment/Akita/TU1-49/2021 (H5N1) | 2021-11-10 | 979/982 (99.69) | M1: 100<br>M2: 98.97 |
| | A/chicken/Kagoshima/B3T/2021 (H5N8) | 2021-02-08 | 978/982 (99.59) | M1: 100<br>M2: 100 |
| Nonstructural protein (NS) | A/hooded crane/Kagoshima/KU-5T/2021 (H5N8) | 2021-11-19 | 837/838 (99.88) | NS1: 100<br>NS2: 100 |
| | A/hooded crane/Kagoshima/KU-d89/2021 (H5N8) | 2021-01-16 | 836/838 (99.76) | NS1: 100<br>NS2: 100 |
| | A/goose/China/1/2021 (H5N8) | 2021-01-29 | 836/838 (99.76) | NS1: 100<br>NS2: 100 |
| | A/goose/Hebei/HG12/2021 (A/H5N8) | 2021-01<br>(Day unknown) | 836/838 (99.76) | NS1: 100<br>NS2: 100 |
| | A/Whooper swan/Sanmenxia/Y12/2020 (H5N8) | 2020-11-08 | 836/838 (99.76) | NS1: 100<br>NS2: 100 |
| | A/chicken/China/2094/2021 (H9N2) | 2021-11-12 | 836/838 (99.76) | NS1: 100<br>NS2: 100 |
| | A/chicken/Kagoshima/B3T/2021 (H5N8) | 2021-11-14 | 836/838 (99.76) | NS1: 100<br>NS2: 100 |
| | A/wild goose/Shandong/SC196/2021 (H5N8) | 2021-01-23 | 834/838 (99.52) | NS1: 99.54<br>NS2: 99.17 |
| | A/Whooper swan/Korea/21WC116/2022 (H5N8) | 2022-01-23 | 834/838 (99.52) | NS1: 99.54<br>NS2: 99.17 |

## LPAIV genes amplification

The PCR mixture was prepared in a final volume of 20 µl containing; 10µl of GoTaq® green master mix (a premixed ready-to-use solution containing GoTaq® DNA polymerase supplied in 2X Green GoTaq® reaction buffer (pH 8.5), 400 µM dATP, 400 µM dGTP, 400 µM dCTP, 400 µM dTTP and 3 mM $MgCl_2$), 1 µl of each 10 µM forward and reverse primers, 7.5 µl of DNase/RNase free water and 0.5 µl of cDNA.

PCR condition: 95$^0$C for 2 minutes of denaturation and 35 cycles of 95$^0$C for 30 seconds, 55$^0$C for 30 seconds, and 72$^0$C for 1 minute followed by a final extension at 72$^0$C for 5 minutes.

**Gel-electrophoresis result of one of the LPAIV (NK12F18-H7N7)**

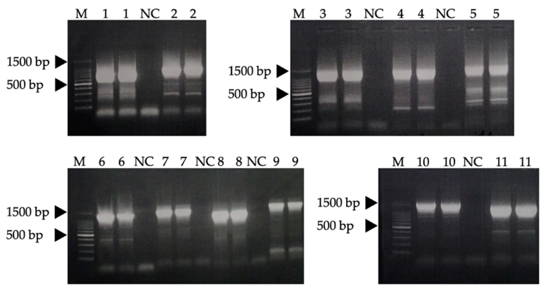


M: DNA ladder (100 bp); 1 (NS, 890 bp); 2 (M, 1027 bp); 3 (NA, 1413 bp), 4 (NP, 1565 bp); 5 (HA, 1778 bp); 6 (PB1a, 1246 bp); 7 (PB1b, 1431 bp); 8 (PAb, 1159 bp); 9 (PAa, 1487 bp); 10 (PB2a, 1473 bp); 11 (PB2b, 1260 bp); NC, negative control. For each gene, 20 μl of PCR reaction was divided into 10 μl and loaded into two wells. The large segments (PB2, PB1, and PA) were divided into two segments for ease of amplification using universal primers designed by Hoffmann *et al*. [27] and primers designed in our laboratory by primer plus 3 program (https://www.primer3plus.com/index.html). The full segment was amplified for HA, NP, NA, NS, and M genes using universal primers designed by Hoffmann *et al*. [27].
